# 682 A Unique Mouth Orthosis for Management of Microstomia

**DOI:** 10.1093/jbcr/iraf019.311

**Published:** 2025-04-01

**Authors:** Michelle Dwertman, Dr Henry Huson, Kelly Spiller

**Affiliations:** University of Cincinnati Medical Center Burn Center; University of Cincinnati Medical Center Burn Center; University of Cincinnati Medical Center Burn Center

## Abstract

**Introduction:**

Microstomia occurs following facial burns with scarring or trauma, causing difficulties with eating, speaking, and oral hygiene. Treatment options vary and orthosis are limited. Some orthoses offer a linear, unidirectional stretch of the mouth opening, lacking verticality and thus the ability to address the oral facial complex of larger facial burns. Secondly, large orthoses are heavy, require dexterity, are hard to sustain a long stretch and thus have decreased adherence. This problem prompted a novel solution: a semirigid oral orthosis made of flexible silicone, lightweight, and able to be sustained independently. The Sequential Compression Appliance to Reduce Scarring (SCARS) oral orthotic provides the benefits of semirigid support with the freedom of being secured internally. It can be customized and progressed in rigidity making it a promising advancement in burn injury care.

**Methods:**

The SCARS oral orthosis is crafted from High Consistency Rubber (HCR) silicone. This silicone layer applies a gentle, consistent pressure while contouring the lips and remaining pliable enough for ease of placement. This material offers customizable thickness options, with greater thickness providing enhanced support and is provided to the patient in graduated options from very flexible/thin to rigid/thick.

Clinical trials were conducted with three patients with severe facial, neck, oral and two with hand burns/scarring resulting in microstomia and hypertrophic scar progression. Over the course of a year, measurements were taken of the oral vertical and horizontal dimensions, including prior to the implementation of the SCARS oral orthosis. In addition, patient preference, eating comfort and overall mouth ROM was assessed via a Likert scale.

**Results:**

In the trials involving three patients with microstomia and facial burns:1. Improved mouth ROM, Eating, and Movement: The patients had significant improvements in eating comfort, and oral ROM. The average Likert scale rating was five. In analyzing mouth opening, there was an average increase of 49% in the horizontal and 131% in the vertical dimensions.2. Patient Preference: There was a clear preference for the SCARS oral orthosis, rating it a five on the Likert scale. Notably, one patient employed its use with a burned hand and contralateral amputation.

**Conclusions:**

The SCARS mouth orthosis is a viable option in the field of burn orthosis, particularly for microstomia. Its innovative blend of flexibility, adaptability, and patient-centric features, holds great promise for individuals dealing with burn-related oral/facial scarring, enhancing comfort and effectiveness of treatment.

**Applicability of Research to Practice:**

This appliance offers increased patient comfort and an option in the management of mouth ROM. Its flexible nature, including its ease of donning, make it a valuable tool for improving scar management outcomes for patients with mouth and combined facial/hand scarring.

**Funding for the Study:**

N/A

